# Effects of spongioplasty on neourethral function following hypospadias repair: an experimental study in rabbits

**DOI:** 10.1590/S1677-5538.IBJU.2019.0453

**Published:** 2020-02-20

**Authors:** Linhai Xie, Yaqi Xi, Xue Zhang, Hongbiao Ding, Senkai Li

**Affiliations:** 1 First Affiliated Hospital of Gannan Medical University Department of Plastic Surgery GanzhouJiangxi China Department of Plastic Surgery, First Affiliated Hospital of Gannan Medical University, Ganzhou, Jiangxi, China; 2 Peking Union Medical College Plastic Surgery Hospital Hypospadias Treatment Center Beijing China Hypospadias Treatment Center, Plastic Surgery Hospital, Peking Union Medical College, Chinese Academy of Medical Sciences, Beijing, China

**Keywords:** Urethra, Fistula, Hypospadias

## Abstract

**Purpose:**

Spongioplasty (mobilization and midline approximation of the two branches of the bifid dysplastic distal corpus spongiosum) can form a covering layer for the neourethra to prevent urethrocutaneous fistula in hypospadias repair surgery. However, it remains unclear whether spongioplasty affects neourethral function. The objective of this study was to compare neourethral function after hypospadias repair with and without spongioplasty.

**Materials and Methods:**

Fourteen congenital hypospadiac New Zealand male rabbits were randomly allocated into two groups, seven animals underwent Duplay hypospadias repair and spongioplasty (experimental group), while seven underwent Duplay surgery alone (control group). Functional differences between groups were assessed by comparing neourethral compliance and flow rate. Two months after surgery, in vivo neourethral compliance was assessed by measuring intraluminal pressure with a digital pressure meter of an isolated neourethral segment, following progressive distension with 1, 2, and 3mL of air. Penises were harvested for uroflowmetry test using a simple device.

**Results:**

Postoperatively, fistula developed in one and zero rabbits in the control and experimental groups, respectively. Mean pressures tended to be higher in the experimental group than in the control group (82.14 vs. 69.57, 188.43 vs. 143.26, and 244.71 vs. 186.29mmHg for 1, 2, and 3mL of air, respectively), but the difference was not statistically significant. Mean flow rates also did not significantly differ between the experimental and control groups (2.93mL/s vs. 3.31mL/s).

**Conclusion:**

In this congenital rabbit model, no obvious functional differences were found between reconstructed urethras after hypospadias repair with and without spongioplasty.

## INTRODUCTION

Urethrocutaneous fistula is one of the most common complications following hypospadias repair. To prevent fistula formation, various tissues (e.g., dartos, de-epithelialized penile skin flap, tunica vaginalis, and corpus spongiosum) have been used as protective layers for the neourethra. Fibrous tissues located on both sides of the urethral plate are known as two branches of the bifid dysplastic corpus spongiosum (in some patients they may persist as healthy, well formed pillars of erectile tissues), in recent years, the use of these tissues in spongioplasty, covering the neourethra alone or with the aid of other tissue layers, has been shown to satisfactorily reduce the fistula rate (1–5). However, the effect of spongioplasty on neourethral function remains unclear. We hypothesized that spongioplasty would cause the neourethra to become thinner and less elastic, due to poor elasticity of the dysplastic spongiosum, resulting in worsened function.

Functional evaluation of the neourethra after hypospadias repair is mainly based on uroflowmetry, more comprehensive assessment requires more complex and intrusive examinations, such as impedance planimetry. However, a simple and effective method has been reported in a rabbit model by Jesus et al., this method measures neourethral compliance based on the intraluminal pressure of an isolated neourethral segment following progressive distension with air using a tensiometer (6). The development of the rabbit penis and urethra are homologous to those processes in humans, and rabbit hypospadias can be induced by 5α-reductase inhibitors (7), thus, in the present study, we evaluated neourethral function following hypospadias repair with and without spongioplasty in a rabbit model of congenital hypospadias.

## MATERIALS AND METHODS

### Rabbit model

A rabbit model of congenital hypospadias was established by feeding finasteride to pregnant New Zealand White rabbits. The study group consisted of 14 male hypospadiac rabbits of similar age (3 months) and weight (2.25-2.65kg), selected from among 22 rabbits with congenital hypospadias. For all rabbits, the urethral meatus was located at the middle third of the penis. All rabbits were kept in individual cages with a standard rabbit diet, water ad libitum, and routine care. The investigation was approved by our university's animal care and use committee (Approval Number: 201631).

### Experimental design

The rabbits were randomly allocated into two equal groups. In experimental group (G1), both Duplay urethroplasty and spongioplasty were per formed, whereas only Duplay urethroplasty was performed in control group (G2). Two months after surgery, rabbits were anesthetized for macroscopic and functional evaluation, they were then sacrificed under general anesthesia and their penises were harvested. All measurements were made by a single investigator (not the surgeon), who was blinded to the grouping of the rabbits.

### Surgical techniques

**Figure 1:**
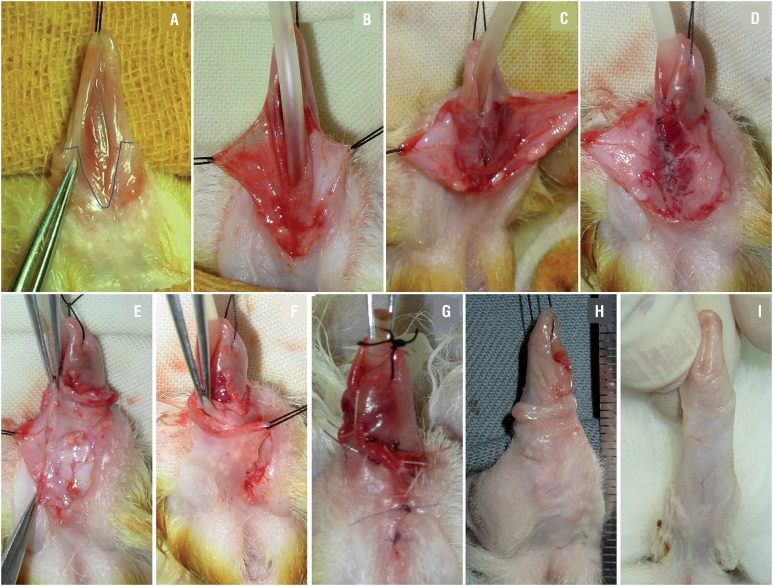
Experimental procedure (Duplay & spongioplasty). **A)** preoperative; **B)** skin dissection; **C)** urethroplasty; **D)** spongioplasty; **E)** de-epithelialized penile skin flap; **F)** skin closure; **G)** operation completed; **H)** two months after surgery; **I)** normal penis of 5 month old male rabbit.

All operations were performed under general anesthesia. In group G1, after classic Duplay urethroplasty over a 10Fr silicone catheter, spongioplasty was performed as follows: two branches of the corpus spongiosum were dissected in a lateral to medial manner from the tunica albuginea carefully, the left branch was flipped toward right and sutured to the right side of the neourethral suture line, while the right branch was flipped and sutured in the opposite manner. Hence, the three suture lines did not overlap. Wound coverage was performed using penile skin flaps on both sides, one flap was partially de-epithelialized and moved across the midline, then sutured under the contralateral skin, such that it served as another waterproof layer to cover the neourethra (Figure-1). In group G2, after urethroplasty was completed, the superficial fascia was sutured to cover the neourethral suture line (this procedure was not performed in Group G1) and spongioplasty was not performed, the remaining procedures were identical to those in group G1. No dressing or stent was left in place in either group.

### Macroscopic evaluation

Under anesthesia, penile and meatal shape were observed, and fistula presence was assessed by injecting water into the urethra while blocking the proximal urethra. After the rabbits had been sacrificed, one animal from each group was perfused with red and blue emulsions via the abdominal aorta and posterior vena cava intubation, respectively. After 24 hours, the penises were harvested for anatomical observation.

### Neourethral compliance examination

Jesus et al. reported a simple and effective method for urethral compliance measurement (6), we completed this part using their method. Under anesthesia and with the urethra in situ, the urethra was ligated at the level of the penile root and a 10Fr silicone catheter was inserted through the meatus, then, the distal urethra was ligated at exactly 3 cm distally from the first ligation, such that an isolated urethral segment was formed (this primarily consisted of neourethra). The catheter tip was placed at the middle of the segment, and its distal end was connected to a digital pressure meter and a syringe through a three-way connector. When air was injected into the segment through the syringe, the intraluminal pressure was transmitted to the pressure meter. The segment was distended with 1, 2, and 3mL of air (with an interval of 5-10 seconds between injections, until the pressure had stabilized), and intraluminal pressures were measured.

### Uroflowmetry

**Figure 2:**
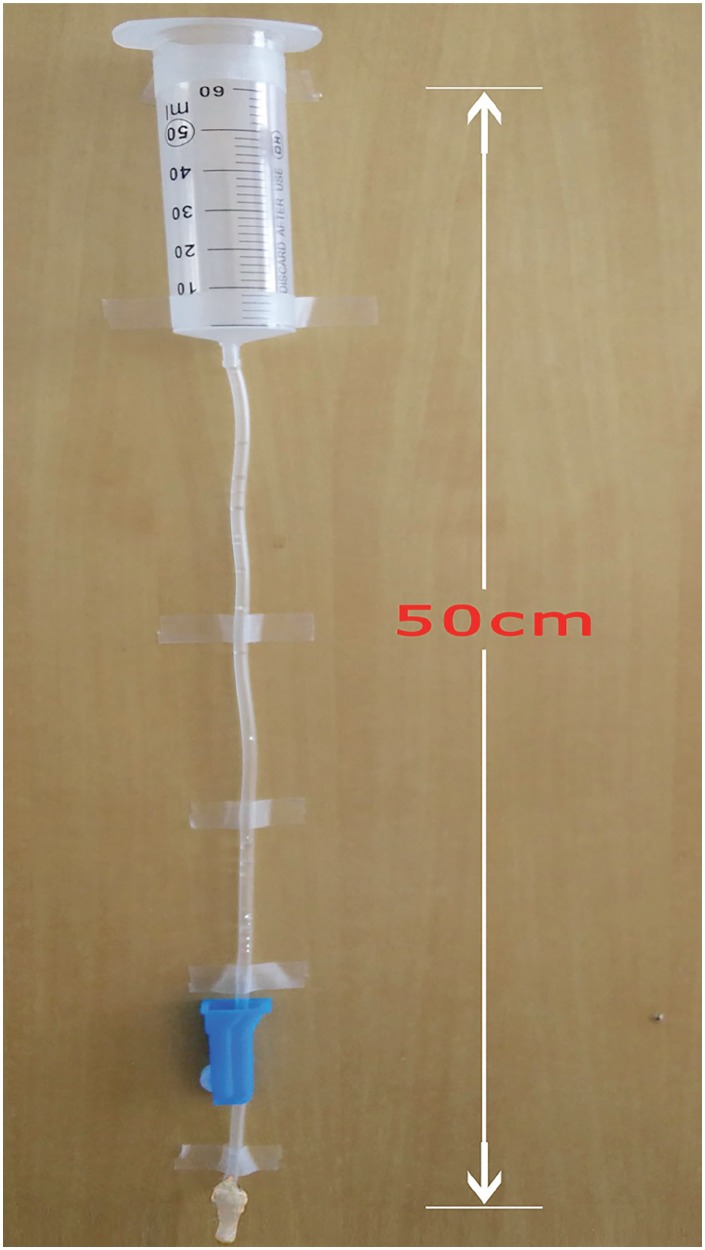
A simple flow rate device.

Uroflowmetry was performed in fresh urethral segments using a passive flow rate protocol developed by Leslie et al. (8). Briefly, a 50mL syringe was connected with an intravenous line, after it had been filled with water, the system was vertically fixed on the wall to form a 50cm water column (Figure-2). After compliance examination, the same 3cm urethral segment was harvested, its proximal end was sutured to a tube and connected to the end of the intravenous line. The mean flow rate was determined by dividing the amount of water (50mL) by the time required to empty the system.

### Statistical analysis

Statistical analysis was performed using SPSS Statistics software (version 17.0, SPSS Inc., Chicago, IL, USA). Data that demonstrated a normal distribution were expressed as the mean and standard deviation. t-tests were used to compare uroflowmetry data between the two groups. Compliance differences between the two groups were compared by analysis of variance of the repeated measurement data. Differences with P <0.05 were considered to be statistically significant.

## RESULTS

### Surgical results

Spongioplasty was successfully performed in each animal, the mean operating time in group G1 was 100min. (90-120min.), while the mean operating time in group G2 was 70min. (50-80min.). All animals survived until the scheduled sacrifice date and voided spontaneously throughout the study period, no signs of infection or flap necrosis were found postoperatively. Mild rupture of the distal ventral incision (approximately 3mm) occurred in one rabbit in group G1, this healed within 2 weeks.

### Macroscopic appearance

**Figure 3:**
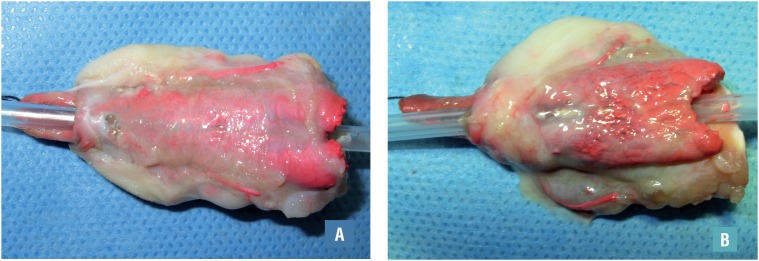
Corpus spongiosum of experimental (A) and control group (B).

The meatus was located in a nearly normal position in all rabbits, except the rabbit with the ruptured incision. The prepuce ring was intact and the urethra could be easily calibrated using a 10Fr catheter, indicating that no obvious strictures had formed. A needle size fistula was found in one rabbit in group G2, located near the meatus. Significant differences were observed in the morphology of the corpus cavernosum between the perfusion specimens of the two groups. In group G1, the ventral side of the neourethra was covered by thick spongiosum tissue, the two branches of the bifurcated urethral spongiosum were nearly integrated, with spongiosum absent only near the meatus. In group G2, there was considerable distance between the two branches, the entire ventral neourethral wall was thin and translucent, and the intraluminal catheter could be observed. (Figure-3)

### Neourethral compliance

All animals were successfully tested and the results are shown in Table-1, intraurethral pressure was proportional to the volume of injected air. The mean pressure of group G1 was slightly higher than that of group G2, but this difference was not statistically significant.

**Table 1:** Intraluminal pressures generated by air injection.

Volume of air	Group	N	Pressure (x±s, mmHg)
	G1	7	82.14±16.77
1mL	G2	7	69.57±13.34
	G1	7	188.43±45.52
2mL	G2	7	143.26±30.55
	G1	7	244.71±61.44
3mL	G2	7	186.29±44.04
			*F*=4.11, P=0.0654

### Uroflowmetry

One rabbit was randomly selected from each group for perfusion and did not undergo uroflowmetry examination. The remaining rabbits were all successfully tested and the results are shown in Table-2, there were no significant differences in uroflowmetry between the two groups.

**Table 2:** Flow rate results.

Group	N	Folw speed(x±s, mL/s)
G1	6	2.93±0.36
G2	6	3.31±0.59
		*t*=1.35, *P*=0.2053

## DISCUSSION

The use of well-vascularized tissue as a protective intermediate layer between the neourethra and the skin is considered an effective measure to prevent urinary fistula, and various tissues (e.g., dartos, de-epithelialized penile skin flap, tunica vaginalis, and corpus spongiosum) have been used (9–12). In 2000, Beaudoin and Yerkes first reported the separation of two branches of the bifurcated urethral spongiosum, which were combined and then used to cover the ventral side of the new urethra, this procedure was termed “spongioplasty” (13, 14). Subsequently, there have been many reports of the use of bifurcated spongiosum (alone or in combination with other tissue layers) as a healthy intermediate tissue layer for use in urethroplasty. It is well-vascularized, robust, and can be conveniently harvested, it is also considered to be physiologically appropriate for use as a protective layer of the neourethra, such that it may aid in the natural propulsion of urine and semen (2, 15, 16). Spongioplasty can reduce suture tension during urethroplasty, and increases the tissue thickness of the ventral penis, thus helping to reduce fistula rate (17). However, it has not yet been reported whether spongioplasty affects neourethral function.

We hypothesized that the combination of two branches of the bifurcated spongiosum may compress or reduce the new urethra due to suture tension, thus modifying its effective diameter, moreover, we hypothesized that this approach may limit urethral expansion because of its fibrous content and poor elasticity, thereby producing unsatisfactory urodynamics. Therefore, we assessed urinary flow rate, urethral diameter, and compliance for comparison of functional differences between experimental and control urethras.

In previous studies, most investigators have employed uroflowmetry to evaluate neourethral function following hypospadias repair; it is considered to be a direct reflection of function, and the flow rate in patients after repair is lower than that in normal controls (8, 18, 19). According to Poiseuille's law (Q=πr^4^ΔP/8μL; where Q=flow, r=radius of pipe, AP=pressure difference, μ=viscosity of liquid, and L=length of pipe), the urethral radius has the greatest impact on flow. We examined isolated urethral segments of equal length, and the pressure difference (AP, 50cm water column), viscosity of water (μ), and urethral length (L) were fixed, urethral diameter was the only variable in the formula. The difference in flow rate reflected the difference in urethral diameter. Because there was no statistical difference in the flow rate between the two groups, we inferred that there was no significant difference in urethral diameter between the two groups.

Compliance describes the resistance of an elastic organ to undergo deformation by an external force, in particular, highly compliant organs require lower pressure to undergo deformation. In a tubular structure such as the urethra, increased compliance and elasticity indicate that the diameter of the urethra is more likely to increase during urination, thus reducing the resistance of flow (R, R=8μL/πr^4^) and increasing the flow rate. Jesus found that the reconstructed urethra has lower compliance than the normal urethra (6), which may explain why many patients have abnormal uroflowmetry results without obvious urethral strictures. We examined the compliance of the neourethra by measuring the intraurethral pressure generated by injection of fixed volumes of air (greater pressure means worse compliance), and found that there was no significant difference in pressure between the two groups. Since the two groups also showed no significant differences in neourethral diameter (10Fr), or in the results of uroflowmetry, we concluded that the difference in compliance between the two groups was not significant.

The development of the rabbit penis and urethra is similar to that observed in humans (e.g., the process by which the urethral plate curls to form the urethra), and the size of the rabbit penis is convenient for surgical manipulation, therefore, the rabbit is an ideal model for the investigation of hypospadias repair. This study used a rabbit model of drug-induced congenital hypospadias, which is superior to the rabbit model of surgically produced hypospadias. Because there are many differences between the normally developed urethra and the hypospadiac urethral plate (e.g., anatomical and biomechanical characteristics, elasticity, and wound healing), which limit the applicability of the surgically produced hypospadias model (20). These differences were especially important in the present study.

No fistulas developed in the experimental group in this study, whereas one rabbit in the control group (14.29%) developed fistula. Postoperative anatomical observation showed the protective effect of spongioplasty on the neourethra. This suggests that spongioplasty may aid in fistula prevention, and has no significant impact on neourethral function (i.e., flow rate and urethral compliance). In addition, we did not evaluate a physiological urethral segment in this study, however, the reunited corpus spongiosum may aid with urethral voiding in vivo.

This study had a few notable limitations. Because rabbits with similar weight and hypo-spadias classification are difficult to obtain, our sample size was relatively small, which may have affected the generalizability of the findings. In addition, uroflowmetry was performed after compliance assessment (due to limited sample size), therefore the tested urethra had been expanded, which inevitably affected the uroflowmetry results, however, urethras of both groups were equally affected, so this may not have greatly influenced our conclusions.

## CONCLUSIONS

In this congenital rabbit model, spongioplasty is surgically feasible and helps prevent fistula, moreover, it had no significant effect on neourethral compliance or flow rate, compared with these parameters in rabbits who had not undergone spongioplasty. Our results suggest that spongioplasty could form an effective protective layer for the neourethra without impairing its function.
